# Ancient harbour infrastructure in the Levant: tracking the birth and rise of new forms of anthropogenic pressure

**DOI:** 10.1038/srep05554

**Published:** 2014-07-03

**Authors:** Nick Marriner, Christophe Morhange, David Kaniewski, Nicolas Carayon

**Affiliations:** 1 CNRS, Laboratoire Chrono-Environnement UMR 6249, Université de Franche-Comté, UFR ST, 16 route de Gray, 25030 Besançon, France; 2 Institut Universitaire de France, Aix-Marseille Université, CEREGE UMR 7330, Europôle de l'Arbois, BP 80, 13545 Aix-en-Provence cedex 04, France; 3 Institut Universitaire de France, EcoLab, UMR 5245 CNRS UPS INPT, Université Paul Sabatier-Toulouse 3, Bâtiment 4R1, 118 Route de Narbonne, 31062 Toulouse cedex 9, France; 4 Department of Archaeology, University of Southampton, Highfield, Southampton SO17 1BF, United Kingdom

## Abstract

Beirut, Sidon and Tyre were major centres of maritime trade from the Bronze Age onwards. This economic prosperity generated increased pressures on the local environment, through urbanization and harbour development. Until now, however, the impact of expanding seaport infrastructure has largely been neglected and there is a paucity of data concerning the environmental stresses caused by these new forms of anthropogenic impacts. Sediment archives from Beirut, Sidon and Tyre are key to understanding human impacts in harbour areas because: (i) they lie at the heart of ancient trade networks; (ii) they encompass the emergence of early maritime infrastructure; and (iii) they enable human alterations of coastal areas to be characterized over long timescales. Here we report multivariate analyses of litho- and biostratigraphic data to probe human stressors in the context of their evolving seaport technologies. The statistical outcomes show a notable break between natural and artificial sedimentation that began during the Iron Age. Three anchorage phases can be distinguished: (i) Bronze Age proto-harbours that correspond to natural anchorages, with minor human impacts; (ii) semi-artificial Iron Age harbours, with stratigraphic evidence for artificial reinforcement of the natural endowments; and (iii) heavy human impacts leading to completely artificial Roman and Byzantine harbours.

As in terrestrial environments^1^, the world's oceans are subject to increasing and often unregulated anthropogenic disturbances^2^. This trend has been exacerbated by a growth in human populations in coastal areas and increasing demands for marine resources^3^. In the Eastern Mediterranean, for example, the Levantine and Egyptian coasts have suffered from rapid and uncontrolled coastal development that has had adverse repercussions upon the environment^4^,^5^ and ecosystems. Amongst these threats, seaport activities are of major concern^6^. The impacts associated with modern harbour construction and activities are diverse and include: (i) shifts in depositional and hydrodynamic regimes as a result of the blocking of littoral currents by protruding structures; (ii) the erosion of down-drift coasts, problems of rapid sedimentation in lagoon inlets, estuaries, and harbour channels; (iii) changing water quality; and (iv) a heightened risk of property loss and damage^7^. Today, several Eastern Mediterranean harbours are still experiencing significant environmental damage, including sites in Lebanon, Israel, Palestine and Egypt^4^,^5^,^8^,^9^.

The Levantine coast is a particularly important region to study when and how the human stressors associated with seaport infrastructure have evolved, because the Eastern Mediterranean is seen as one of the cradles of ancient maritime technology, acting as a natural communications link for the major cultural centres of the Levant, Cyprus, Crete, Greece and North Africa^10^ (Fig. 1). Furthermore, its maritime harbourworks were many and varied and transgress all time periods from the Bronze Age up to present day, suggesting the gradual rise of human-modified coastal environments.

In these coastal areas, human modification of sedimentary patterns and processes - through urban development and the expansion of maritime infrastructure - has led to an alteration of natural sedimentary environments and the creation of new depositional patterns articulated around man-made structures^11^. These alterations (e.g. geochemical pollution, urban waste, changes in natural coastal processes and shifts in sediment supply) underscore the key role of human activities in driving environmental changes at the land-sea interface^12^,^13^. Ancient Mediterranean harbours comprise one of the best examples for this type of human-induced modification, with well-identified modifications in sediment patterns, geochemical signatures^14^,^15^,^16^ and the creation of unique strata of anthropogenic origin^17^. Based on present knowledge, these processes are first recorded in the Eastern Mediterranean, where the earliest archaeological evidence for artificial maritime structures dates to the Middle/Late Bronze Age^18^. Amongst the early man-made structures identified, submerged boulder piles at the Middle Bronze Age site of Yavne-Yam (southern Mediterranean coast of Israel) suggest premeditated human enterprise to improve the quality of the natural anchorage (Marcus, personal communication). At Atlit (Carmel coast, Israel), the first well-dated Phoenician mole has been attributed to around 900 BCE^19^, during the Iron Age.

Since the Bronze Age, there has been a great diversity of harbour infrastructure in coastal areas, reflecting a plethora of human modifications in line with evolving technologies, leading to dramatic palaeoenvironmental changes that have played out at a variety of temporal and spatial scales^20^,^21^. Nonetheless, understanding these technological changes and the evolution of human pressures is challenging because many ancient harbour sites have not yet been identified or are today buried beneath city centres^22^,^23^,^24^, or have been reworked or even destroyed.

Here we use statistical analyses of litho- and biostratigraphic data to track the birth and rise of new forms of human pressures on the environment, linked to the emergence of the harbours of Beirut, Sidon and Tyre (coastal Lebanon; Fig. 2). Increased stratigraphic and palaeoecological stresses associated with expanding man-made structures and the evolution of Levantine harbour technology since the Bronze Age, are used to probe early impacts of human societies at the land-sea interface. Beirut, Sidon and Tyre are particularly important due to the great antiquity of their maritime vocation^25^ and their sediments have preserved the imprints of some of the earliest coastal harbourworks in the Mediterranean.

## Results

We performed multivariate statistical analyses on litho- and biostratigraphic data from ancient harbour cores from Beirut, Sidon and Tyre (Figs. 2 and 3). These sediment archives have allowed the chronostratigraphy and palaeogeographical evolution^26^ of the three basins to be reconstructed since the Holocene marine transgression^27^,^28^,^29^. The data matrix comprises 85 entries: 19 for Beirut, 30 for Sidon and 36 for Tyre. For the past 8000 years, four main groups are identified by the different statistical analyses (Figs. 4,5,6): (i) a ‘Transgressive lagoon' group; (ii) a ‘Pocket beach' group; (iii) an ‘Artificial harbour' group; and (iv) a ‘Harbour apogee' group. Three of these four periods correspond to active harbour phases and mirror the increasing weight of human impacts on the Lebanese coastal area. The hierarchical clustering analyses confirm that the litho- and biostratigraphic assemblages are distinct with a series of primary breaks in the dendrogram consistent with the different harbour phases.

Sixteen points constitute group 1, the ‘Transgressive lagoon' cluster. Brackish species and silts consistent with natural low-energy environments dominate this group. The unit is dated to between 8000 and 6000 cal. years BP. Group 2 comprises 27 points and corresponds to the ‘Pocket beach' cluster. The biostratigraphy is characterized by coastal species, and sands and gravels for the lithostratigraphy. The decline in brackish species and fine-grained facies translates higher energy dynamics indicative of the Maximum Marine Ingression after 6000 cal. years BP. Eighteen points are clustered in Group 3 which corresponds to the ‘Artificial harbour phase' broadly dating to the 1^st^ millennium BCE. Marine lagoonal taxa and fine-grained sands dominate the unit. Group 4 is made-up of 24 points and is consistent with the ‘Harbour apogee'. This cluster is dominated by lagoonal taxa and fine-grained sediments, and corresponds to the Roman and Byzantine periods. For some entries, there is a strong gravel fraction comprising numerous ceramic, seeds and wood fragments in addition to an important shell fraction, typical of ancient harbour depositional environments^26^. At Tyre, a sub-branch of this final group corresponds to the ‘Harbour abandonment' phase, characterized by coastal ostracod taxa and sand facies indicative of a shift to higher energy dynamics.

The Neighbour Joining analysis (Fig. 7) clearly separates two main clusters by a sharp statistical break in the median part of the dendrogram. The first cluster encompasses samples corresponding to low human impacts on the environment (i.e. the ‘Transgressive lagoon' and ‘Pocket beach' units). By contrast, the second cluster clearly shows a break between natural and artificial environments, revealing high anthropogenic disturbances at the sea-land interface (the ‘Artificial harbour' and ‘Harbour apogee' units). This major statistical break is further highlighted by the PCA (Fig. 8) where axis 1 (that explains the highest amount of the total variance) clearly separates these two main clusters, and also provides a distinct discrimination between each group forming the clusters. In the Neighbour Joining analysis and in the PCA, the shift from natural to artificial sedimentary systems is particularly transparent (Figs. 7 and 8).

## Discussion

Port basins constitute unique archives of human intervention in the coastal zone and the resulting environmental changes^26^. Recent geoarchaeological investigations have allowed the archaeological community to move beyond the purely architectural aspects of harbourworks, and set them within the wider environmental context. Research has demonstrated that the sediment records from the ancient harbours of Beirut, Sidon and Tyre are particularly rare and precious because they clearly demonstrate that human intervention in the coastal environment, through harbour infrastructure, gradually evolved in a number of technological steps that left clear anthropogenic signatures in the geological archives^18^,^27^,^28^,^29^ (Fig. 3). The shifts in the litho- and biostratigraphy associated with harbour deposits directly translate changes in the degree of basin protection, often characterized by a rapid accumulation of differently-sized sediments following a sharp fall in water competence brought about by artificial harbourworks. These artificial changes engendered major ecological shifts in the harbour basins^30^, translated by discontinuities in the biostratigraphic suites. Our multivariate analyses reveal that the human pressures leading to evolutionary/technological transitions are clearly expressed by statistical breaks in the cluster dendrograms (Figs. 4 to 6).

Although differences are observed in the sediment deposits, there are patterns in both the vertical (stratigraphic) and lateral evolutions of the three basins, changing from natural coves to increasingly artificialized seaports that mirror urban and infrastructure developments^17^. The added value of these new multivariate analyses is to quantitatively frame each palaeoenvironmental phase and precisely identify the onset of major human impacts in harbour environments. Four stages have been elucidated.

### Phase 1: 8000 to 6000 years ago – before direct human interference

At all three sites, the first phase identified by the hierarchical clustering analyses corresponds to transgressive lagoon systems between 8000 and 6000 years ago, in a context of rising post-glacial sea level. The marine flooding surface is dated to ~8000 cal. years BP, coherent with the onset of clastic coast sedimentation throughout the *circum* Mediterranean^31^,^32^. The molluscan and ostracod assemblages are dominated by species with lagoonal affinities, indicative of relatively low-energy marginal marine environments. This period corresponds to the Early Holocene Humid Phase^33^,^34^ and we infer a ponding of freshwater runoff at Beirut, Sidon and Tyre, behind partially drowned sandstone ridges that produced the brackish water conditions.

### Phase 2: proto-harbour phase – low human impacts

The second cluster identified by the numerical analyses marks the transition to coastal conditions at the end of the Holocene marine transgression, around 6000 years ago. Stabilization of Mediterranean sea level at this time^35^,^36^ promoted a sedenterisation of human societies along present coastlines. In the Levant, coastal settlements were preferentially founded around low-energy coastal basins and the mouths of fluvial systems^37^,^38^,^39^,^40^. Coastal sands, and marine-lagoonal and coastal fauna consonant with semi-protected pocket beach systems characterize the litho- and biostratigraphies of the harbour basins during this phase. One of the Levantine coast's defining geomorphological traits is the series of south-north trending Pleistocene shorelines that rim tracts of the region's seaboard^41^. Referred to as ‘kurkar' in Israel and ‘ramleh' in the Lebanon, these sandstone ridges have created a unique coastal geomorphology exploited by human societies for many millennia. At numerous localities on the Phoenician coastline, Holocene flooding of the shore-parallel ridges has given rise to small islands and islets, many of which served as anchorage havens during the Bronze Age, including for instance Dor, Arwad and Ras Ibn Hani^18^,^39^,^42^,^43^,^44^. For several millennia after 6000 years cal. BP, the three basins evolved as semi-protected pocket beaches that would have been particularly attractive to coastal populations in the context of expanding maritime trade during the Bronze Age^45^,^46^,^47^. Throughout this early phase, the harbours of the three Bronze Age settlements coincided with areas of partially drowned sandstone with very little need for human modification of the original geomorphological endowments. Beirut, Sidon and Tyre, established during the 3^rd^ millennium BCE, all integrate partially drowned islets into their harbour models. The natural defences are translated stratigraphically by medium to fine-grained sands that began accreting after 6000 cal. BP, and are clearly differentiated from the preceding lagoonal phase.

A lack of diagnostic harbour clays is consonant with absent or modest harbourworks. During this proto-harbour phase, a simple ‘lighter' system would have been utilized. Boats from this period were generally small and hauled from the water onto the beachface. Larger trade vessels were anchored in semi-protected bays and pocket coves, typical of Beirut, Sidon and Tyre's geomorphological configuration at this time, and goods were ferried to and from the shoreline by lighter craft. Offshore anchoring is corroborated by finds of Bronze Age stone anchors at Sidon and Tyre, concurrent with the extensive discoveries from Byblos^18^.

Towards the end of the Middle Bronze Age and early Late Bronze Age, archaeological evidence attests to the onset of human modification of these natural anchorages (e.g.^38^). With the expansion of Bronze Age trade, the Levant's major ports of call had to be safe in all seasons, including facilities for docking, repair, maintenance and entrepot. A rich literature shows that early engineers carved installations out of the sheltered landward side of rocky outcrops^37^,^38^,^48^. Although this infrastructure is notoriously difficult to date, a Bronze Age origin has been attributed to it by some scholars^49^,^50^.

On stratigraphic grounds, the existence of such artificial harbourworks at Beirut, Sidon and Tyre has been difficult to prove unequivocally. Only at Sidon have we been able to date a moderate shift from medium to finer-grained sedimentary conditions during the Middle Bronze Age (~1500 cal. BCE^27^). At Beirut and Tyre, there is a paucity of stratigraphic evidence for Bronze Age harbourworks. This suggests the moderate stratigraphic impacts of these edifices. A dearth of diagnostic harbour units at Byblos^51^,^52^, the pearl of the Phoenician coast during the Bronze Age^53^, further corroborates the findings from Beirut, Sidon and Tyre. The difficulties presented by maritime engineering at this time appear to have been overcome by the extensive use of lighter vessels to load and unload larger trade crafts anchored offshore^54^.

From this first harbour phase, therefore, it is noted that Beirut, Sidon and Tyre, in addition to the other major Bronze Age ports of the Levantine coast, coincided with areas of partially drowned sandstone. Natural anchorages were the rule with very little need for human modification of the original geomorphological endowments^18^.

### Phase 3: towards artificial harbour basins and pronounced human impacts

Archaeological evidence from the three sites attests to a pattern of expanding Mediterranean trade that prompted coastal populations into artificializing the basins during the Iron Age. Beirut, Sidon and Tyre were key players in a cabotage network running from Gaza in the south up to Arwad in the north. There is a clear spatial pattern in the distribution of the Levant's anchorages, with intercalating distances of ~20 to 40 km between each site. Seafaring on the Phoenician coast during the Bronze and Iron Ages was essentially dominated by short hopping-type navigation^55^,^56^.

The increased use of iron at the turn of the 1^st^ millennium BCE meant that much larger shipping vessels could be constructed^57^. Such boats had greater requirements than their Bronze Age counterparts, with needs for docking, repair and entrepot. Archaeological evidence from Athlit, a small Phoenician trading outpost 55 km south of Tyre, attests to an artificial mole that has been dated to the 9^th^/8^th^ centuries BCE^43^. Iron Age III/Persian quays at Beirut are also consistent with this model of well-developed port infrastructure during the Iron Age^58^.

Clearly dated fine-grained silts and clays are manifest during the following Hellenistic and Roman periods at all three sites, suggesting an increased human alteration of the natural environment. These are well identified by the hierarchical clustering analyses, with a clear break in the dendrograms of the litho- and biostratigraphic data translating the transition from natural to artificial coastal sedimentation (Fig. 7). Although this is not the technological apogee of the seaport trio, the numerical analyses concur advanced harbour infrastructure.

Rapid rates of silting threatened the long-term viability of the harbours, culminating in repeated dredging of the basins^59^. Elsewhere in the Mediterranean, the transition to Roman rule is marked by the refashioning of many ancient harbours^60^. Although at some sites completely artificial roadsteads were carved or annexed onto the coastline, the enduring maritime trade routes meant that the major ports of call changed very little^61^. Instead, the Romans chose to reinforce the pre-existing ports and in many cases significantly remoulded harbour morphologies. Although there is very little architectural evidence pertaining to these port overhauls at Beirut, Sidon and Tyre, stratigraphic hiatuses and widespread dating inversions nonetheless point to considerable changes. The three settlements were all key cities of the Levantine seaboard at this time, requiring effective harbour management strategies for trade and transport purposes.

### Phase 4: Harbour apogee – heavy human impacts

The consolidation of Roman construction techniques, coupled with the economic importance of the Levantine seaboard during the Byzantine period, is translated by lagoon-like harbours very well protected from the open sea. Biostratigraphically, the late Roman-Byzantine apogee is marked by a sharp increase in lagoonal species at all three sites, consistent with hyposaline basins (Figs. 4,5,6). At no other point in the stratigraphic record does one observe such well-protected harbours. Artificial Romano-Byzantine moles have been elucidated at both Sidon and Tyre, their construction greatly facilitated by the discovery of hydraulic concrete^48^,^62^,^63^,^64^,^65^.

At Beirut and Sidon, the period is translated by a diagnostic plastic harbour clay consistent with heavily-modified coastal environments^14^. An analogous unit is observed at Tyre, although here the facies comprises fine-grained silts and sands. This human-induced shift in the sedimentary environment is clearly identified by the hierarchical clustering analyses. Biostratigraphically, the late Roman-Byzantine apogee is marked by a sharp increase in *Cyprideis torosa* at all three sites, consistent with hyposaline basins. Lagoonal and fine-grained macrofauna assemblages also characterize these facies. At no other point in the stratigraphic record are such well-protected ports observed. The consolidation of Roman construction techniques, coupled with the economic importance of the Levantine seaboard during the Byzantine period, culminated in lagoon-like harbours very well protected from the open sea.

The Levant was arguably one of the richest regions of the Byzantine Empire during the early centuries of its existence^66^. It was at this time that the area witnessed a demographic upswing, corroborated by settlement expansion, urban growth and agricultural development, suggesting significant and continuous human pressures on the environment. The material record supports a broad picture of rural settlement and rich agricultural production in the communities of Greater Syria until the mid-6^th^ century CE^67^. This apogee is clearly recorded in the harbour stratigraphy.

Our work has demonstrated that the 6^th^ to 7^th^ centuries CE marked a turning point in the evolution of the three harbours. Coarse-grained sand units attest to a semi-abandonment of the harbours and silting up of the basins' landward fringes, underpinned by a plethora of cultural, economic and natural factors. We stress that this demise was relative. From a technological apogee during the late Roman and Byzantine periods, a medievalization of the three cities is observed along the lines of the Arabic medinas.

## Conclusion

The Levantine coast is a key geographical region to characterize the full range of responses to human modification of coastal areas. For the ancient harbours of Beirut, Sidon and Tyre, the increased human pressure, brought about by technological innovations, is well documented by shifts in the litho- and biostratigraphic data. Three types of anchorages were distinguished according to a range of human pressures on the coastal environment. (i) *Proto-harbours*. Bronze Age societies exploited coastal geomorphology to establish natural anchorage havens in low-energy coves. The impacts of these harbours on the environment were very limited. (ii) *Semi-artificial Iron Age harbours*. This harbour type lies at the intersection between natural anchorages and completely artificial harbours. The type stratigraphy comprises early human modification of the coastal environment, expressed by a fine-grained sand/silt facies indicative of the artificial reinforcement of shielding ridges. (iii) *Roman and Byzantine artificial harbours*. Natural roadsteads were no longer a prerequisite for port foundation. At Beirut, Sidon and Tyre this period is marked by a significant phase of coastal artificialization and port remodelling. The type sediment sequences are characterized by litho- and biostratigraphical signatures that are similar at all three sites (Fig. 7). We stress that human activities linked to the harbour and urban developments, have mediated Levantine coastal areas since the Bronze Age, with an amplification of environmental damage during the Roman and Byzantine periods. This study, using stratigraphy and multivariate statistics, highlights the impacts of expanding harbour infrastructure and new technologies on the environmental dynamics of the Levantine coast.

## Methods

### Litho- and biostratigraphic data

**Figure 1:**
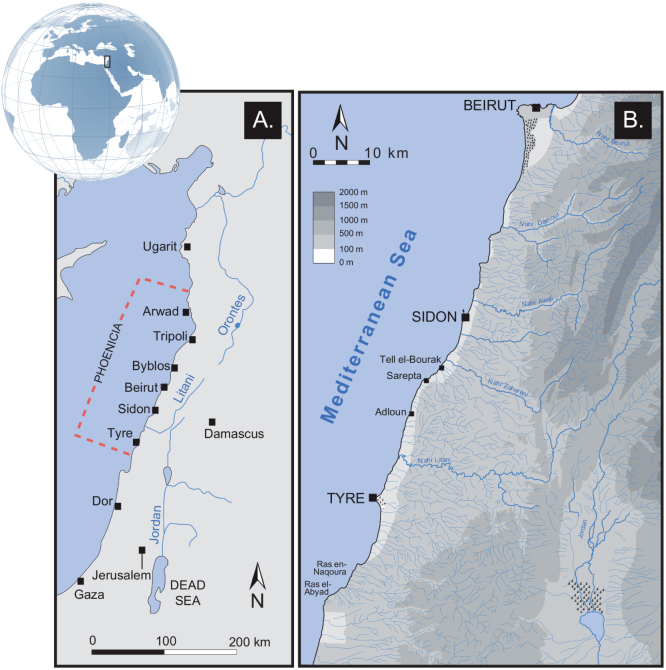
(A) Levant maritime façade. (B) Phoenician coast depicting Beirut, Sidon and Tyre (drawn by N. Carayon, Adobe Illustrator).

**Figure 2:**
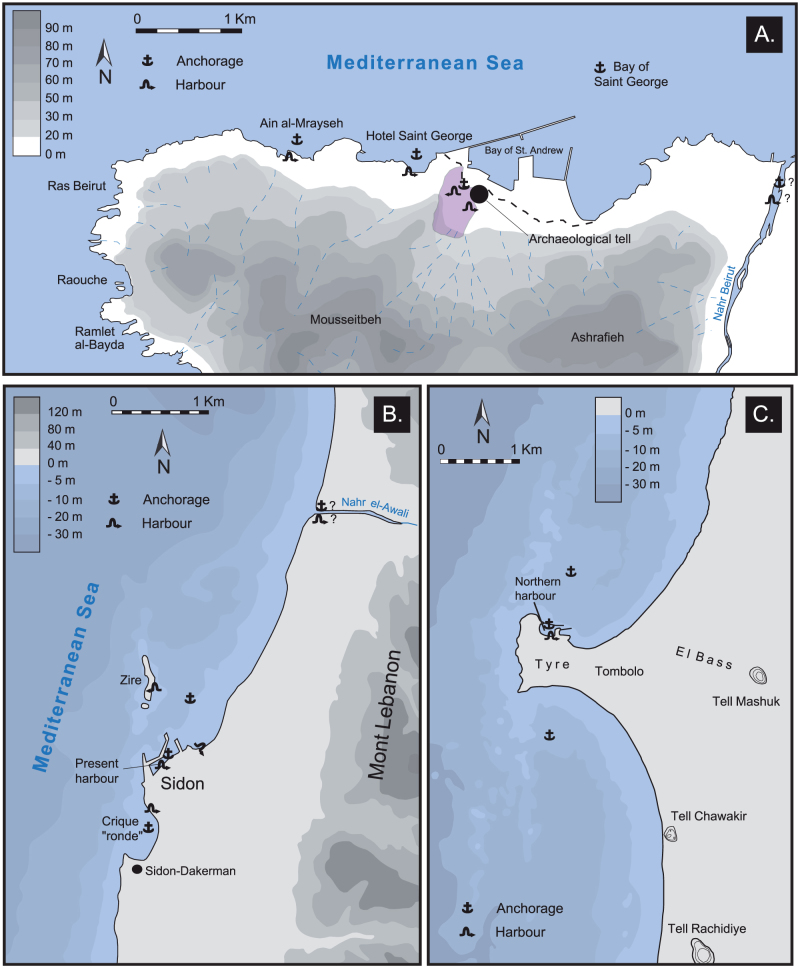
Location maps of (A) Beirut; (B) Sidon; and (C) Tyre (drawn by N. Carayon, Adobe Illustrator).

**Figure 3:**
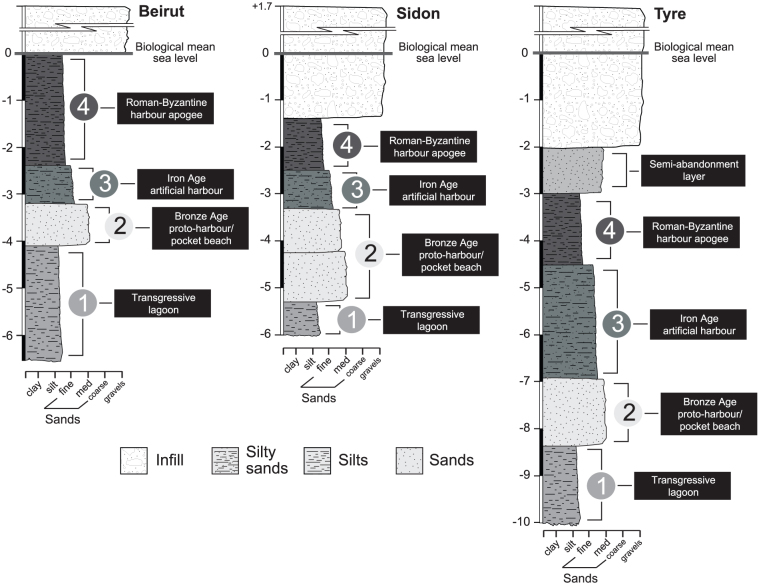
Type stratigraphy of the ancient harbours of Beirut, Sidon and Tyre.

The geoarchaeology of Beirut, Sidon and Tyre's ancient harbours has been documented by a series of studies^18^,^27^,^28^,^29^ (Figs. 1 and 2). These investigations focussed upon the use of chronostratigraphy to derive palaeogeographical reconstructions since the early to mid-Holocene marine transgression. It has been demonstrated that the heart of the ancient basins lie beneath the present city centres and that the surface areas of the harbours were >50% than present day. Nonetheless, these early studies lacked quantified analyses of the various sedimentological and palaeoecological datasets using multivariate statistics to probe, for instance, early human impacts in coastal areas. To fill this knowledge gap, we selected three well-dated type cores^27^,^28^,^29^, one from each of these ancient harbours, to investigate when and how harbour technologies have evolved since the Bronze Age (Fig. 3). We refer the reader to reference^68^ for details on the palaeogeography of the ancient harbour basins. Sediment cores were sub-sampled at a resolution of 10 to 50 cm, depending on the nature of the facies. In the laboratory, samples were oven dried at 40°C and subsequently wet sieved to separate out the gravels (>2 mm), sands and silts fractions. Malacological material was picked from the gravels fraction and identified using references^69^,^70^,^71^. Species were assigned to ecological groups based on^72^,^73^. Part of the sand fraction was dry sieved to establish various grain-size parameters (see^17^). The non-sieved part was used for ostracod analyses. We extracted at least 100 tests from each sample. Ostracods were identified and assigned to ecological assemblages based on^74^. Four groups were recognized: lagoonal, marine-lagoonal, coastal and marine. Previous studies have demonstrated that ostracods are particularly sensitive recorders of natural and human-induced changes at the land-sea interface^75^,^76^,^77^. All macro- and microfauna data in the samples were counted and are expressed as a percentage of total tests. The litho- and biostratigraphic data were normalized and tabulated into data matrices of equal lengths. We subsequently performed a suite of statistical analyses, both individually for each harbour basin and collectively, in order to differentiate the evolution of the harbour phases.

### Numerical analyses

**Figure 4:**
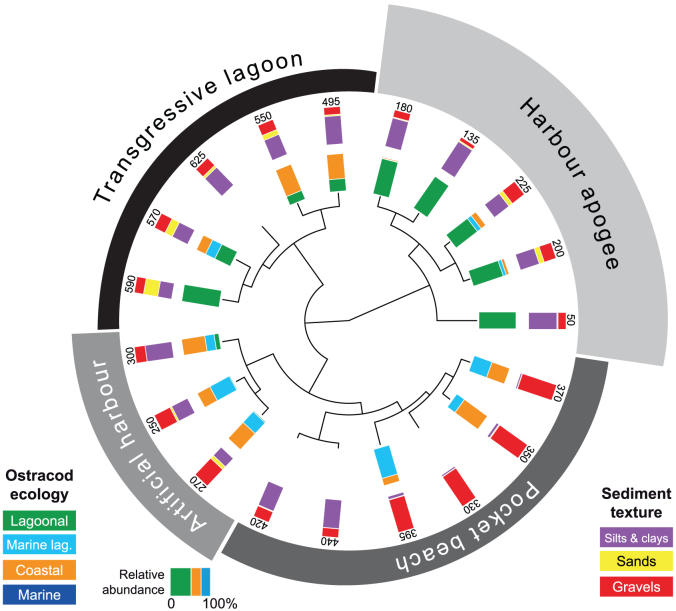
Radial dendrogram of the litho- and biostratigrphic data from a type core drilled in the ancient harbour of Beirut. The different evolutionary phases are clearly differentiated by the statistical breaks in the dendrogram, evolving from a natural basin to a heavily artificialised seaport during the Roman period. The numbers denote the stratigraphic depth of each sample below Mean Sea Level (MSL).

**Figure 5:**
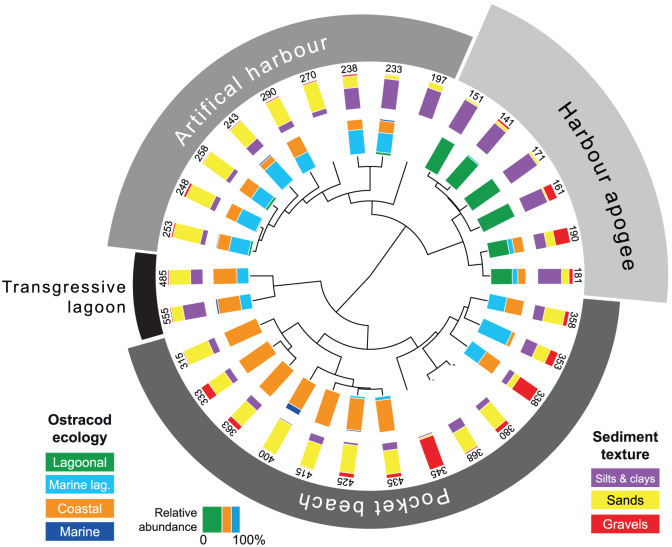
Radial dendrogram of the litho- and biostratigraphic data from a type core drilled in the ancient harbour of Sidon. The numbers denote the stratigraphic depth of each sample below MSL.

**Figure 6:**
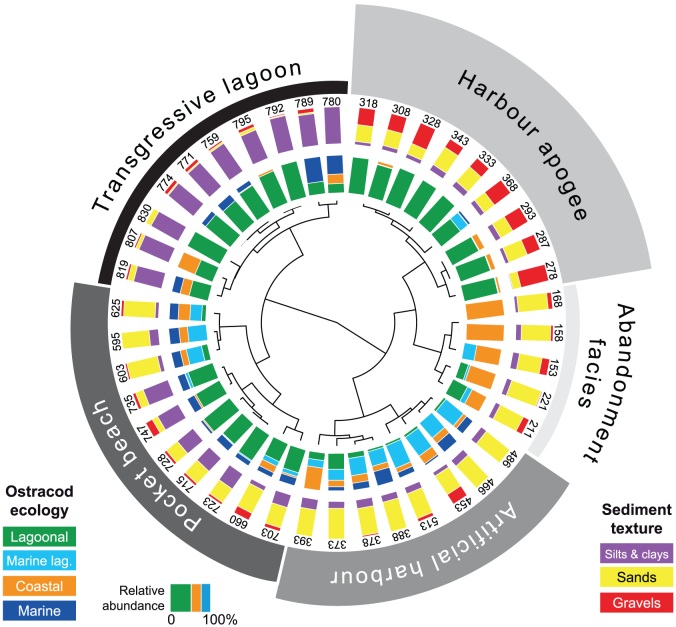
Radial dendrogram of the litho- and biostratigrphic data from a type core deriving from the ancient northern harbour of Tyre. The numbers denote the stratigraphic depth of each sample below MSL.

**Figure 7:**
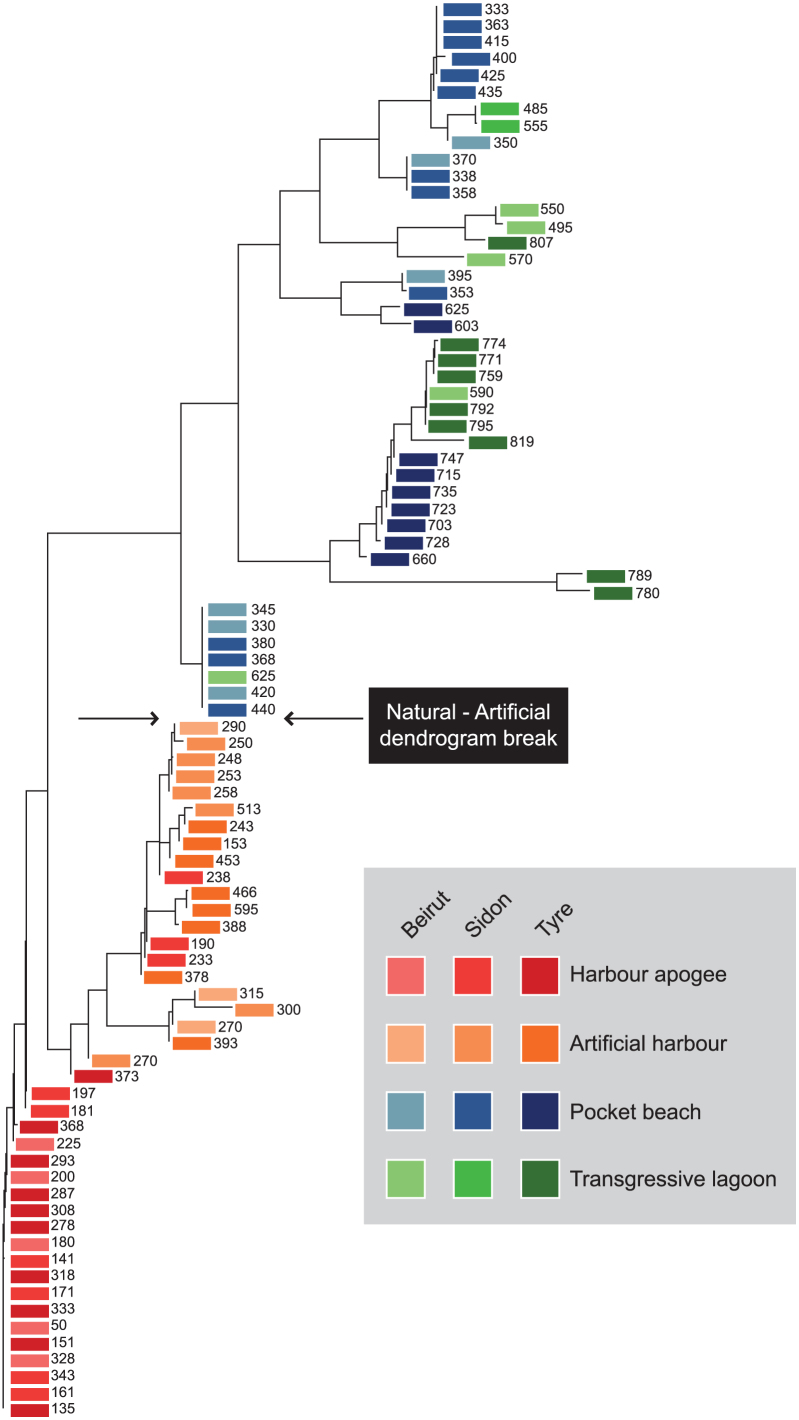
Neighbour-joining cluster analysis of the litho- and biostratigraphic data from the ancient harbours of Beirut, Sidon and Tyre. The transition from natural to artificial environments is highlighted by the arrows. The numbers denote the stratigraphic depth of each sample below MSL.

**Figure 8:**
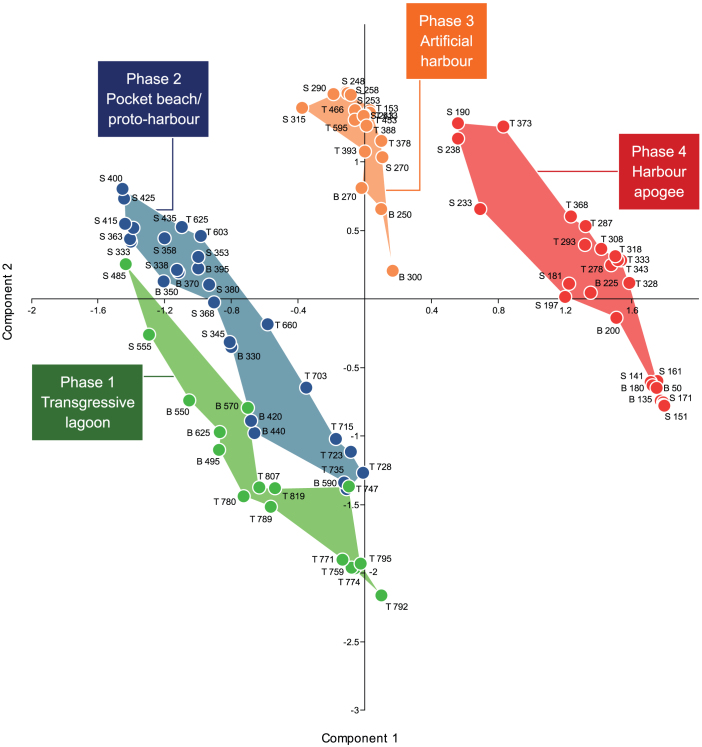
PCA analysis of the stratigraphic data (var-covar matrix). Components 1 & 2 explain 67% of the variation in the data. Key species on PC1 notably include *Cyprideis torosa* (lagoonal) and, on PC2, marine lagoonal and coastal species. The numbers denote the stratigraphic depth of each sample below MSL. The letters B, S and T are consistent with samples from Beirut, Sidon and Tyre respectively.

Litho- and biostratigraphic data were analyzed using hierarchical clustering, Neighbour-Joining cluster analysis (NJ) and Principal Components Analysis (PCA). In this instance, these multivariate analyses are effective because they allow the data to be studied across multiple dimensions while taking into account the effects of all variables on the responses. Such statistical techniques are particularly pertinent because they reduce the complexity of the large dataset and allow a representation of the underlying structure of the data to be generated. They allow patterns not necessarily evident in the initial stratigraphic data to be identified. A number of recent studies have underscored the importance of multivariate techniques in probing coastal sediment archives from the Levantine seaboard^78^,^79^ but such methods have never been applied to ancient harbour contexts. In this study, the hierarchical clustering was used to group data from a variety of chronostratigraphic contexts and temporal scales, by creating a cluster tree or dendrogram. We used radial dendrograms to clearly display the groups formed by the analysis. The radial dendrogram is a multilevel hierarchy, where clusters at one level are joined as clusters to the next level (Figs. 4,5,6). The NJ method is an alternative technique for hierarchical cluster analysis, finding hierarchical groupings in multivariate data sets. Here, it is based on litho- and biostratigraphic data (presence/absence and abundance). NJ analysis was used to compute the lengths of tree branches, using branches as distances between groups of data (descending type). NJ was computed using correlation as the similarity measure and final branch as the root (Fig. 7). A PCA was also performed to test the ordination of samples by assessing major changes in litho- and biostratigraphic data. The main variance is loaded by the PCA-Axes 1 and 2 (Fig. 8). We found that the statistical analyses are particularly effective in linking diagnostic coastal stratigraphies with harbour technology and establishing the early environmental impacts of the infrastructure.

## Author Contributions

N.M. undertook the litho- and biostratigraphical analyses. D.K. contributed analytical tools. N.M., C.M., D.K. and N.C. analyzed and interpreted the data. N.C. produced Figs. 1 and 2. N.M. produced Figs. 3 to 8. N.M., C.M., D.K. and N.C. wrote the paper.
